# Supplementary material for: Diabetes Mellitus and Dental Implants: A Systematic Review and Meta-Analysis
Source: Materials (Basel). 2022 Apr 29;15(9):3227. doi: 10.3390/ma15093227 (PMC9105616; doi:10.3390/ma15093227)
Supplement: Supplementary file 1 [file materials-15-03227-s001.zip › materials-1658118-supplementary.pdf]

SUPPLEMENTARY MATERIAL

a. Dental implant-related journals included in the manual search;
b. Reference list of the included studies;
c. Table S1. Detailed data of the included studies;
d. Table S2. Quality assessment of the included studies, according to the National Institutes of Health (NIH).

a. Dental implant-related journals included in the manual search

Clinical Implant Dentistry and Related Research, Clinical Oral Implants Research, European Journal of Oral Implantology, Implant Dentistry, International Journal of Implant Dentistry, International Journal of Oral and Maxillofacial Implants, International Journal of Oral Implantology, International Journal of Prosthodontics, Journal of Clinical Periodontology, Journal of Oral Implantology, Journal of Periodontology, Journal of Prosthetic Dentistry, Journal of Prosthodontics, Journal of Prosthodontic Research.

b. Reference list of the included studies

1. Accursi GE. Treatment outcomes with osseointegrated Brånemark implants in diabetic patients: a retrospective study [thesis]. Toronto (ON): University of Toronto; 2000.
2. Aguilar-Salvatierra A, Calvo-Guirado JL, González-Jaranay M, Moreu G, Delgado-Ruiz RA, Gómez-Moreno G. Peri-implant evaluation of immediately loaded implants placed in esthetic zone in patients with diabetes mellitus type 2: a two-year study. Clin Oral Implants Res. 2016 Feb;27(2):156-61.
3. Alqahtani F, Alqhtani N, Alkhtani F, Devang Divakar D, Al-Kheraif AA, Javed F. Clinicoradiographic markers of peri-implantitis in cigarette-smokers and never-smokers with type 2 diabetes mellitus at 7-years follow-up. J Periodontol. 2020 Sep;91(9):1132-1138.
4. Alsaadi G, Quirynen M, Komárek A, van Steenberghe D. Impact of local and systemic factors on the incidence of late oral implant loss. Clin Oral Implants Res. 2008b Jul;19(7):670-6.
5. Alsaadi G, Quirynen M, Michiles K, Teughels W, Komárek A, van Steenberghe D. Impact of local and systemic factors on the incidence of failures up to abutment connection with modified surface oral implants. J Clin Periodontol. 2008a Jan;35(1):51-7.
6. Al-Sabbagh M, Thomas MV, Bhavsar I, De Leeuw R. Effect of Bisphosphonate and Age on Implant Failure as Determined by Patient-Reported Outcomes. J Oral Implantol. 2015;41(6):e287-e291.
7. Alsahhaf A, Alshiddi IF, Alshagroud RS, Al-Aali KA, Vohra F, Abduljabbar T. Clinical and radiographic indices around narrow diameter implants placed in different glycemic-level patients. Clin Implant Dent Relat Res. 2019 Aug;21(4):621-626.
8. Altay MA, Tozoğlu S, Yıldırımyan N, Özarslan MM. Is History of Periodontitis a Risk Factor for Peri-implant Disease? A Pilot Study. Int J Oral Maxillofac Implants. 2018 January/February;33(1):152–160.
9. Anner R, Grossmann Y, Anner Y, Levin L. Smoking, diabetes mellitus, periodontitis, and supportive periodontal treatment as factors associated with dental implant survival: a long-term retrospective evaluation of patients followed for up to 10 years. Implant Dent. 2010 Feb;19(1):57-64.
10. Atarchi AR, Miley DD, Omran MT, Abdulkareem AA. Early Failure Rate and Associated Risk Factors for Dental Implants Placed With and Without Maxillary Sinus Augmentation: A Retrospective Study. Int J Oral Maxillofac Implants. 2020 Nov/Dec;35(6):1187-1194.
11. Bell CL, Diehl D, Bell BM, Bell RE. The immediate placement of dental implants into extraction sites with periapical lesions: a retrospective chart review. J Oral Maxillofac Surg. 2011 Jun;69(6):1623-7.

12. Boardman N, Darby I, Chen S. A retrospective evaluation of aesthetic outcomes for single-tooth implants in the anterior maxilla. Clin Oral Implants Res. 2016;27(4):443-451.
13. Boboeva O, Kwon TG, Kim JW, Lee ST, Choi SY. Comparing factors affecting dental-implant loss between age groups: A retrospective cohort study. Clin Implant Dent Relat Res. 2021 Apr;23(2):208-215.
14. Cabrera-Domínguez J, Castellanos-Cosano L, Torres-Lagares D, Machuca-Portillo G. A Prospective Case-Control Clinical Study of Titanium-Zirconium Alloy Implants with a Hydrophilic Surface in Patients with Type 2 Diabetes Mellitus. Int J Oral Maxillofac Implants. 2017 Sep/Oct;32(5):1135-1144.
15. Cannizzaro G, Leone M. Restoration of partially edentulous patients using dental implants with a microtextured surface: a prospective comparison of delayed and immediate full occlusal loading. Int J Oral Maxillofac Implants. 2003;18(4):512-522.
16. Chang LC. Risk factors associated with early failure of maxillary versus mandibular implants: A retrospective study. Int J Oral Implantol (Berl). 2020;13(1):55-63.
17. Chrcanovic BR, Kisch J, Albrektsson T, Wennerberg A. Factors Influencing Early Dental Implant Failures. J Dent Res. 2016;95(9):995-1002.
18. Clauser C, Sforza NM, Menini I, Kalemaj Z, Buti J; Collaborators of Accademia Toscana di Ricerca Odontostomatologica (ATRO) IPI Group. Immediate Postextraction Single-Tooth Implants and Provisional Crowns in the Esthetic Area: 2-year Results of a Cohort Prospective Multicenter Study- Patient-Centered Outcomes. Int J Oral Maxillofac Implants. 2020 Jul/Aug;35(4):833-840.
19. Coskunses FM, Tak Ö. Clinical performance of narrow-diameter titanium-zirconium implants in immediately loaded fixed full-arch prostheses: a 2-year clinical study. Int J Implant Dent. 2021 Apr 16;7(1):30.
20. Daneshvar SS, Matthews DC, Michuad PL, Ghiabi E. Success and Survival Rates of Dental Implants Restored at an Undergraduate Dental Clinic: A 13-Year Retrospective Study with a Mean Follow-up of 5.8 Years. Int J Oral Maxillofac Implants. 2016 Jul-Aug;31(4):870-5.
21. Daubert DM, Weinstein BF, Bordin S, Leroux BG, Flemming TF. Prevalence and predictive factors for peri-implant disease and implant failure: a cross-sectional analysis. J Periodontol. 2015 Mar;86(3):337-47.
22. Dhanrajani PJ, Al-Rafee MA. Single-tooth implant restorations: a retrospective study. Implant Dent. 2005;14(2):125-130.
23. Dowell S, Oates TW, Robinson M. Implant success in people with type 2 diabetes mellitus with varying glycemic control: a pilot study. J Am Dent Assoc. 2007;138:355-361.
24. Doyle SL, Hodges JS, Pesun IJ, Baisden MK, Bowles WR. Factors affecting outcomes for single-tooth implants and endodontic restorations. J Endod. 2007 Apr;33(4):399-402.
25. Erdogan Ö, Uçar Y, Tatlı U, Sert M, Benlidayı ME, Evlice B. A clinical prospective study on alveolar bone augmentation and dental implant success in patients with type 2 diabetes. Clin Oral Implants Res. 2015;26(11):1267-1275.
26. Feher B, Lettner S, Heinze G, Karg F, Ulm C, Gruber R, Kuchler U. An advanced prediction model for postoperative complications and early implant failure. Clin Oral Implants Res. 2020 Oct;31(10):928-935.
27. French D, Larjava H, Ofec R. Retrospective cohort study of 4591 Straumann implants in private practice setting, with up to 10-year follow-up. Part 1: multivariate survival analysis. Clin Oral Implants Res. 2015;26(11):1345-1354.
28. Gherlone EF, Capparé P, Tecco S, Polizzi E, Pantaleo G, Gastaldi G, Grusovin MG. Implant Prosthetic Rehabilitation in Controlled HIV-Positive Patients: A Prospective Longitudinal Study with 1-Year Follow-Up. Clin Implant Dent Relat Res. 2016 Aug;18(4):725-34.
29. Ghiraldini B, Conte A, Casarin RC, et al. Influence of Glycemic Control on Peri-Implant Bone Healing: 12-Month Outcomes of Local Release of Bone-Related Factors and Implant Stabilization in Type 2 Diabetics. Clin Implant Dent Relat Res. 2016;18(4):801-809.

30. Gjelvold B, Kisch J, Mohammed DJH, Chrcanovic BR, Albrektsson T, Wennerberg A. Immediate Loading of Single Implants, Guided Surgery, and Intraoral Scanning: A Nonrandomized Study. Int J Prosthodont. 2020 Sep/Oct;33(5):513-522.
31. Gómez-Moreno G, Aguilar-Salvatierra A, Rubio Roldán J, Guardia J, Gargallo J, Calvo-Guirado JL. Peri-implant evaluation in type 2 diabetes mellitus patients: a 3-year study. Clin Oral Implants Res. 2015;26(9):1031-1035.
32. Göthberg C, André U, Gröndahl K, Thomsen P, Slotte C. Bone Response and Soft Tissue Changes Around Implants With/Without Abutments Supporting Fixed Partial Dentures: Results From a 3-Year, Prospective, Randomized, Controlled Study. Clin Implant Dent Relat Res. 2016;18(2):309-322.
33. Grandi T, Guazzi P, Samarani R, Garuti G, Grandi G. Immediate loading of two unsplinted implants retaining the existing complete mandibular denture in elderly edentulous patients: 1-year results from a multicentre prospective cohort study. Eur J Oral Implantol. 2012 Spring;5(1):61-8.
34. Grandi T, Guazzi P, Samarani R, Garuti G. Immediate positioning of definitive abutments versus repeated abutment replacements in immediately loaded implants: effects on bone healing at the 1-year follow-up of a multicentre randomised controlled trial. Eur J Oral Implantol. 2012 Spring;5(1):9-16.
35. Grandi T, Guazzi P, Samarani R, Grandi G. Immediate loading of four (all-on-4) post-extractive implants supporting mandibular cross-arch fixed prostheses: 18-month follow-up from a multicentre prospective cohort study. Eur J Oral Implantol. 2012;5(3):277-285.
36. Grandi T, Guazzi P, Samarani R, Maghaireh H, Grandi G. One abutment-one time versus a provisional abutment in immediately loaded post-extractive single implants: a 1-year follow-up of a multicentre randomised controlled trial. Eur J Oral Implantol. 2014 Summer;7(2):141-9.
37. Grandi T, Guazzi P, Samarani R, Grandi G. A 3-year report from a multicentre randomised controlled trial: immediately versus early loaded implants in partially edentulous patients. Eur J Oral Implantol. 2013 Autumn;6(3):217-24.
38. Han J, Tang Z, Zhang X, Meng H. A prospective, multi-center study assessing early loading with short implants in posterior regions. A 3-year post-loading follow-up study. Clin Implant Dent Relat Res. 2018 Feb;20(1):34-42.
39. He J, Zhao B, Deng C, Shang D, Zhang C. Assessment of implant cumulative survival rates in sites with different bone density and related prognostic factors: an 8-year retrospective study of 2,684 implants. Int J Oral Maxillofac Implants. 2015;30(2):360-371.
40. Higuchi K, Rosenberg R, Davó R, Albanese M, Liddelow G. A Prospective Single-Cohort Multicenter Study of an Innovative Prefabricated Three-Implant-Supported Full-Arch Prosthesis for Treatment of Edentulous Mandible: 1-year Report. Int J Oral Maxillofac Implants. 2020 Jan/Feb;35(1):150-159.
41. Ji TJ, Kan JY, Rungcharassaeng K, Roe P, Lozada JL. Immediate loading of maxillary and mandibular implant-supported fixed complete dentures: a 1- to 10-year retrospective study. J Oral Implantol. 2012;38 Spec No:469-476.
42. Kappel S, Giannakopoulos NN, Eberhard L, Rammelsberg P, Eiffler C. Immediate Loading of Dental Implants in Edentulous Mandibles by Use of Locator® Attachments or Dolder® Bars: Two-Year Results from a Prospective Randomized Clinical Study. Clin Implant Dent Relat Res. 2016;18(4):752-761.
43. Keller EE, Tolman DE, Eckert SE. Maxillary antral-nasal inlay autogenous bone graft reconstruction of compromised maxilla: a 12-year retrospective study. Int J Oral Maxillofac Implants 1999;14:707–721.
44. Kim S, Jung UW, Cho KS, Lee JS. Retrospective radiographic observational study of 1692 Straumann tissue-level dental implants over 10 years: I. Implant survival and loss pattern. Clin Implant Dent Relat Res. 2018 Oct;20(5):860-866.

45. Klotz AL, Ott L, Krisam J, Schmitz S, Seyidaliyeva A, Rammelsberg P, Zenthöfer A. Short-term performance of implant-supported restorations fitted in general dental practice: A retrospective study. Int J Oral Maxillofac Implants. 2019 September/October;34(5):1169–1176.
46. Koka S, Babu NM, Norell A. Survival of dental implants in post-menopausal bisphosphonate users. J Prosthodont Res. 2010;54(3):108-111.
47. Kourtis SG, Sotiriadou S, Voliotis S, Challas A. Private practice results of dental implants. Part I: survival and evaluation of risk factors--Part II: surgical and prosthetic complications. Implant Dent. 2004;13(4):373-385.
48. Krennmair G, Seemann R, Weinländer M, Krennmair S, Piehslinger E. Clinical outcome and peri-implant findings of four-implant-supported distal cantilevered fixed mandibular prostheses: five-year results. Int J Oral Maxillofac Implants. 2013;28(3):831-840.
49. Krennmair S, Hunger S, Forstner T, Malek M, Krennmair G, Stimmelmayr M. Implant health and factors affecting peri-implant marginal bone alteration for implants placed in staged maxillary sinus augmentation: A 5-year prospective study. Clin Implant Dent Relat Res. 2019 Feb;21(1):32-41.
50. Krennmair S, Weinländer M, Malek M, Forstner T, Krennmair G, Stimmelmayr M. Mandibular Full-Arch Fixed Prostheses Supported on 4 Implants with Either Axial Or Tilted Distal Implants: A 3-Year Prospective Study. Clin Implant Dent Relat Res. 2016;18(6):1119-1133.
51. Le BT, Follmar T, Borzabadi-Farahani A. Assessment of short dental implants restores with single-unit nonsplinted restorations. Implant Dent. 2013 Oct;22(5):499-502.
52. Lee KJ, Cha JK, Sanz-Martin I, Sanz M, Jung UW. A retrospective case series evaluating the outcome of implants with low primary stability. Clin Oral Implants Res. 2019 Sep;30(9):861-871.
53. Levin L, Ofec R, Grossmann Y, Anner R. Periodontal disease as a risk for dental implant failure over time: a long-term historical cohort study. J Clin Periodontol. 2011 Aug;38(8):732-7.
54. Lobato RPB, Kinalski MA, Martins TM, Agostini BA, Bergoli CD, Dos Santos MBF. Influence of low-level laser therapy on implant stability in implants placed in fresh extraction sockets: A randomized clinical trial. Clin Implant Dent Relat Res. 2020 Jun;22(3):261-269.
55. Loo W, Jin LJ, Cheung MNB, Wang M. The impact of diabetes on the success of dental implants and periodontal healing. African Journal of Biotechnology 2009;8:5122-5127.
56. Malchiodi L, Balzani L, Cucchi A, Ghensi P, Nocini PF. Primary and Secondary Stability of Implants in Postextraction and Healed Sites: A Randomized Controlled Clinical Trial. Int J Oral Maxillofac Implants. 2016;31(6):1435-1443.
57. Maló P, de Araújo Nobre M, Gonçalves Y, Lopes A. Long-Term Outcome of Implant Rehabilitations in Patients with Systemic Disorders and Smoking Habits: A Retrospective Clinical Study. Clin Implant Dent Relat Res. 2016;18(4):649-665.
58. Maló P, de Araújo Nobre M, Lopes A, Ferro A, Botto J. The All-on-4 treatment concept for the rehabilitation of the completely edentulous mandible: A longitudinal study with 10 to 18 years of follow-up. Clin Implant Dent Relat Res. 2019 Aug;21(4):565-577.
59. Maló P, de Araújo Nobre M, Lopes A, Ferro A, Nunes M. The All-on-4 concept for full-arch rehabilitation of the edentulous maxillae: A longitudinal study with 5-13 years of follow-up. Clin Implant Dent Relat Res. 2019 Aug;21(4):538-549.
60. Malo P, de Araújo Nobre M, Lopes A, Moss SM, Molina GJ. A longitudinal study of the survival of All-on-4 implants in the mandible with up to 10 years of follow-up. J Am Dent Assoc. 2011;142(3):310-320.
61. Mijiritsky E, Mazor Z, Lorean A, Levin L. Implant diameter and length influence on survival: interim results during the first 2 years of function of implants by a single manufacturer. Implant Dent. 2013;22(4):394-398.
62. Morales-Vadillo R, Leite FP, Guevara-Canales J, et al. Retrospective study of the survival and associated risk factors of wedge-shaped implants. Int J Oral Maxillofac Implants. 2013;28(3):875-882.

63. Morris HF, Ochi S, Winkler S. Implant survival in patients with type 2 diabetes: placement to 36 months. Ann Periodontol. 2000 Dec;5(1):157-65.
64. Niedermaier R, Stelzle F, Riemann M, Bolz W, Schuh P, Wachtel H. Implant-Supported Immediately Loaded Fixed Full-Arch Dentures: Evaluation of Implant Survival Rates in a Case Cohort of up to 7 Years. Clin Implant Dent Relat Res. 2017;19(1):4-19.
65. Nogueira TE, Aguiar FMO, de Barcelos BA, Leles CR. A 2-year prospective study of single-implant mandibular overdentures: Patient-reported outcomes and prosthodontic events. Clin Oral Implants Res. 2018 Jun;29(6):541-550.
66. Norton MR. The Influence of Low Insertion Torque on Primary Stability, Implant Survival, and Maintenance of Marginal Bone Levels: A Closed-Cohort Prospective Study. Int J Oral Maxillofac Implants. 2017 Jul/Aug;32(4):849-857.
67. Omran MT, Miley DD, McLeod DE, Garcia MN. Retrospective assessment of survival rate for short endosseous dental implants. Implant Dent. 2015;24(2):185-191.
68. Park SH, Song YW, Sanz-Martín I, Cha JK, Lee JS, Jung UW. Clinical benefits of ridge preservation for implant placement compared to natural healing in maxillary teeth: A retrospective study. J Clin Periodontol. 2020 Mar;47(3):382-391.
69. Ravidà A, Barootchi S, Tattan M, Saleh MHA, Gargallo-Albiol J, Wang HL. Clinical outcomes and cost effectiveness of computer-guided versus conventional implant-retained hybrid prostheses: A long-term retrospective analysis of treatment protocols. J Periodontol. 2018 Sep;89(9):1015-1024.
70. Ravidà A, Tattan M, Askar H, Barootchi S, Tavelli L, Wang HL. Comparison of three different types of implant-supported fixed dental prostheses: A long-term retrospective study of clinical outcomes and cost-effectiveness. Clin Oral Implants Res. 2019 Apr;30(4):295-305.
71. Romandini M, Cordaro M, Donno S, Cordaro L. Discrepancy between patient satisfaction and biologic complication rate in patients rehabilitated with overdentures and not participating in a structured maintenance program after 7 to 12 years of loading. Int J Oral Maxillofac Implants. 2019 September/October;34(5):1143–1151.
72. Romero JLR, Ortiz Garcia I, Jiménez Guerra A, Matos Garrido N, España López A, Monsalve Guil L et al . El tratamiento con implantes en pacientes con diabetes. Un estudio comparativo a 7 años. Av Odontoestomatol. 2020; 36(2):81-88.
73. Rosen PS, Sahlin H, Seemann R, Rosen AS. A 1-7 year retrospective follow-up on consecutively placed 7-mm-long dental implants with an electrowetted surface. Int J Implant Dent. 2018 Aug 23;4(1):24.
74. Saridakis SK, Wagner W, Noelken R. Retrospective cohort study of a tapered implant with high primary stability in patients with local and systemic risk factors-7-year data. Int J Implant Dent. 2018 Dec 17;4(1):41.
75. Schoenbaum TR, Moy PK, Aghaloo T, Elashoff D. Risk Factors for Dental Implant Failure in Private Practice: A Multicenter Survival Analysis. Int J Oral Maxillofac Implants. 2021 Mar-Apr;36(2):388-394.
76. Schwartz-Arad D, Ofec R, Eliyahu G, Ruban A, Sterer N. Long Term Follow-Up of Dental Implants Placed in Autologous Onlay Bone Graft. Clin Implant Dent Relat Res. 2016;18(3):449-461.
77. Shibuya Y, Takeuchi Y, Asai T, Takeuchi J, Suzuki H, Komori T. Maxillary sinus floor elevation combined with a vertical onlay graft. Implant Dent. 2012;21(2):91-96.
78. Sicilia A, Gallego L, Sicilia P, Mallo C, Cuesta S, Sanz M. Crestal bone loss associated with different implant surfaces in the posterior mandible in patients with a history of periodontitis. A retrospective study. Clin Oral Implants Res. 2021 Jan;32(1):88-99.
79. Simons WF, De Smit M, Duyck J, Coucke W, Quirynen M. The proportion of cancellous bone as predictive factor for early marginal bone loss around implants in the posterior part of the mandible. Clin Oral Implants Res. 2015 Sep;26(9):1051-9.

80. Souza CSV, Ortega-Lopes R, Barreno AC, de Moraes M, Albergaria-Barbosa JR, Nóia CF. Analysis of the Survival of Dental Implants Installed in Reconstructed Maxilla With Autogenous Iliac Crest Graft: 7- to 9-Year Follow-Up. J Oral Implantol. 2019 Dec;45(6):427-436.
81. Stacchi C, Troiano G, Rapani A, Lombardi T, Sentineri R, Speroni S, Berton F, Di Lenarda R. Factors influencing the prevalence of peri-implantitis in implants inserted in augmented maxillary sinuses: A multicenter cross-sectional study. J Periodontol. 2021 Aug;92(8):1117-1125.
82. Tattan M, Puranam M, Comnick C, McBrearty C, Xie XJ, Caplan DJ, Avila-Ortiz G, Elangovan S. Surgery start time and early implant failure: A case-control study. Clin Oral Implants Res. 2021 Jul;32(7):871-880.
83. Tawil G, Younan R, Azar P, Sleilati G. Conventional and advanced implant treatment in the type II diabetic patient: surgical protocol and long-term clinical results. Int J Oral Maxillofac Implants. 2008 Jul-Aug;23(4):744-52.
84. Temmerman A, Keestra JA, Coucke W, Teughels W, Quirynen M. The outcome of oral implants placed in bone with limited bucco-oral dimensions: a 3-year follow-up study. J Clin Periodontol. 2015 Mar;42(3):311-8.
85. Troiano G, Luongo R, Romano DC, Galli M, Ravidà A, Wang HL, Laino L. Comparison of immediate versus delayed implant placement in a failed implant site: A retrospective analysis of early implant survival. Int J Oral Implantol (Berl). 2021 Mar 16;14(1):67-76.
86. van Steenberghe D, Jacobs R, Desnyder M, Maffei G, Quirynen M. The relative impact of local and endogenous patient-related factors on implant failure up to the abutment stage. Clin Oral Implants Res. 2002 Dec;13(6):617-22.
87. Wang J, Lerman G, Bittner N, Fan W, Lalla E, Papapanou PN. Immediate versus delayed temporization at posterior single implant sites: A randomized controlled trial. J Clin Periodontol. 2020 Oct;47(10):1281-1291.
88. Werbelow L, Weiss M, Schramm A. Long-term follow-up of full-arch immediate implant-supported restorations in edentulous jaws: a clinical study. Int J Implant Dent. 2020 Jul 30;6(1):34.
89. Zumstein T, Sennerby L. A 1-Year Clinical and Radiographic Study on Hydrophilic Dental Implants Placed with and without Bone Augmentation Procedures. Clin Implant Dent Relat Res. 2016;18(3):498-506.

c. Table S1. Detailed data of the included studies.

Study	Year	Study design	Country / Setting	Patients (male/ female) (n)	Patients' Age Range (mean) (years)	Prosthetic loading	Diabetes type I/II (number patients) / Blood sugar level (HbA1c) verified?	Implant location	Implant used	Were there smokers in the group of patients? [a]
**Accursi**	2000	RA (unicenter)	Canada / University	45 (NM)	15-83 (56)	NM	I (2) and II (13) / NM	Mx, Md	Turned (Brånemark, Nobel Biocare, Göteborg, Sweden)	Smokers: 53.3% (G1), 31.6% (G2)
**Aguilar-Salvatierra**	2016	CCT (unicenter)	Spain / University	85 (44/41)	NM (59)	Immediate	NM (52) / Yes	Mx	Bone Level (Straumann, Basel, Switzerland)	No
**Alqahtani**	2020	RA (unicenter)	Saudi Arabia / University	101 (101/0)	NM (52)	NM	II (50) / Yes	Mx, Md	NM	51 smokers
**Alsaadi (1)**	2008	CCT (unicenter)	Belgium / University	283 (96/187)	18-86 (56.2)	Loading was not applied	I (NM) and II (NM) / NM	Mx, Md	Mk III TiUnite (Nobel Biocare, Göteborg, Sweden)	Yes, but exact number not informed
**Alsaadi (2)**	2008	RA (unicenter)	Belgium / University	412 (172/240)	NM	NM	I (1) and II (9) / NM	Mx, Md	Turned and TiUnite Mk III (Brånemark, Nobel Biocare, Göteborg, Sweden)	61 smokers
**Al-Sabbagh**	2015	RA (unicenter)	USA / University	415 (174/241)	NM (59.4)	NM	II (43) / NM	Mx, Md	NM	46 smokers
**Alsahhaf**	2019	RA (unicenter)	Saudi Arabia / University	119 (76/43)	33-58 (43-52)	NM	II (38) and pre-diabetic (41) / Yes	Mx, Md	NM	No
**Altay**	2018	RA (unicenter)	Turkey / University	13 (6/7)	41-70 (55.2)	Delayed (3-6 mo)	NM (5) / NM	Mx, Md	Several (BEGO, Biotech Dental, Bredent Medical, Straumann)	No
**Anner**	2010	RA (unicenter)	Israel / Private practice	475 (176/299)	NM (52)	NM	NM (49) / NM	Mx, Md	NM	63 smokers
**Atarchi**	2020	RA (multicenter)	USA / University	1343 (516/827)	18-96 (61.7)	NM	NM (52) / Yes	Mx	Several (Astra EV OsseoSpeed, NobelReplace, Biomet 3i, Straumann, Zimmer)	58 smokers
**Bell**	2011	RA (unicenter)	USA / Private	655 (NM)	NM (59)	Delayed (3 mo)	NM	Mx, Md	SLA (Straumann, Waldenburg, Switzerland)	Yes, but exact number not

			practice							informed
**Boardman**	2016	RA (unicenter)	Australia / University	98 (21/77)	23-81 (51.2)	NM	NM (1) / NM	Mx	Several (Nobel Biocare, Straumann, AstraTech, Biomet 3i)	7 smokers
**Boboeva**	2021	RA (unicenter)	South Korea / University	1295 (584/711)	15-86 (46.7, 71.4)	Delayed	NM (72) / NM	Mx, Md	Several (Astra Tech, AB, Mölndal, Sweden; Dentis, Daegu, South Korea; Dentium, Seoul, South Korea; Straumann, Basel, Switzerland; Megagen Implant, Gyeongsan, South Korea; Osstem Implant, Seoul, South Korea).	78 smokers
**Cabrera-Domingue z**	2017	CCT (unicenter)	Spain / University	29 (12/17)	NM (55.9)	Delayed (2 mo)	II (15) / Yes	Mx, Md	SLActive (Straumann, Basel, Switzerland)	NM
**Cannizzaro**	2003	CCT (unicenter)	Italy / Private practice	28 (14/14)	18-72 (38)	Immediate, delayed	II (2) / NM	Mx, Md	Microtextured (Spline Twist implants, Centerpulse Dental, Carlsbad, USA)	6 smokers
**Chang**	2020	RA (unicenter)	China / Hospital	376 (222/154)	NM (49)	Delayed	NM (19) / NM	Mx, Md	Osseotite, Certain Taper (Biomet 3i, Palm Beach Gardens, USA)	15 smokers<br>52 former smokers
**Chrcanovic**	2016	RA (unicenter)	Sweden / Public service	2670 (NM)	17-90 (54.1)	Immediate, delayed	I (28) and II (101) / No	Mx, Md	Several (Brånemark turned, Nobel TiUnite, Nobel Active, Astra TiOblast/Osseospeed, Straumann SLA, Bego Semados, XiVE Friadent)	474 smokers<br>47 former smokers
**Clauser**	2020	PS (multicenter)	Italy / Private practice	214 (92/122)	17-84 (48.3)	Immediate	NM (3) / NM	Mx, Md	NanoTite Certain Tapered (Biomet 3i)	24 heavy smokers<br>4 extra-heavy smokers
**Coskunses**	2021	PS (unicenter)	Turkey / University	28 (17/11)	23-72 (52)	Immediate	NM (2) / NM	Mx, Md	Roxolid Bone Level Tapered (Straumann, Basel, Switzerland)	6 smokers
**Daneshvar**	2016	RA (unicenter)	Canada / University	111 (40/71)	17-86 (56.1)	NM	NM (21) / NM	Mx, Md	NM	8 smokers

**Daubert**	2015	RA (unicenter)	USA / University	96 (48/48)	31-86 (67.6)	Immediate, delayed	NM (5) / NM	Mx, Md	Several	7 smokers
**Dhanrajani**	2005	RA (unicenter)	Saudi Arabia / Private practice	101 (30/71)	17-69 (35.4)	Immediate, delayed	NM (15) / Yes	Mx, Md	Several (Brånemark, 3i, Calcitek Sulzer, Steri-Oss)	17 light smokers 7 heavy smokers
**Dowell**	2007	CCT (unicenter)	USA / University	35 (18/17)	29-81 (NM)	Loading was not applied	II (25) / Yes	Mx, Md	SLA (Straumann, Basel, Switzerland)	No
**Doyle**	2007	RA (unicenter)	USA / University	171 (NM)	NM (47.5)	NM	I (1) and II (2) / NM	Mx, Md	NM	10 smokers
**Erdogan**	2015	CCT (multicenter)	Turkey / University	24 (12/12)	NM (51)	Delayed (4 mo)	II (12) / NM	Mx	SLA (Straumann, Waldenburg, Switzerland)	No
**Feher**	2020	RA (unicenter)	Austria / University	1132 (505/627)	NM (50.6)	Evaluation of implants before loading	NM (33) / NM	Mx, Md	NM	157 light smokers 60 heavy smokers
**French**	2015	RA (unicenter)	Canada / Private practice	2060 (922/1138)	15-85 (50.6)	Immediate, delayed	I (4) and II (23) / NM	Mx, Md	Sandblasted and acid-etched (SLA, Straumann, Waldenburg, Switzerland)	29 heavy smokers (>15 cig./day)
**Gherlone**	2016	PS (unicenter)	Italy / Hospital	68 (22/46)	40-73 (55.3)	Delayed (2-3 mo)	NM (31) / NM	Mx, Md	WinSix (BioSAFin., Ancona, Italy)	29 light smokers 13 heavy smokers
**Ghiraldini**	2016	CCT (unicenter)	Brazil / University	51 (28/23)	37-70 (51.6-56.4)	Delayed (4 mo)	II (32) / Yes	Md	NM (SIN, São Paulo, Brazil)	No
**Gjelvold**	2020	PS (unicenter)	Sweden / Public service	46 (25/21)	NM (40)	Immediate	NM (2) / NM	Mx	Tapered Internal (BioHorizons, Birmingham, USA)	3 smokers
**Gómez-Moreno**	2015	CCT (multicenter)	Spain / University	67 (33/34)	NM (59-63)	Delayed (3-5 mo)	II (46) / Yes	Mx	SLA (Straumann, Waldenburg, Switzerland)	No
**Grandi (1)**	2012	CCT (multicenter)	Italy / Private practice	47 (22/25)	52-78 (62.3)	Immediate	NM (2) / NM	Md	JDEvolution (JDentalCare, Modena, Italy)	11 light and heavy smokers
**Grandi (2)**	2012	RCT (multicenter)	Italy / Private	28 (11/17)	39-64 (51)	Immediate	NM (1) / NM	Mx, Md	JDEvolution (JDentalCare, Modena, Italy)	9 light smokers

			practice							
**Grandi (3)**	2012	PS (multicenter)	Italy / Private practice	42 (13/29)	71-89 (76.5)	Immediate	NM (2) / NM	Md	JDEvolution (JDentalCare, Modena, Italy)	9 light smokers
**Grandi (4)**	2013	RCT (multicenter)	Italy / Private practice	80 (31/49)	39-65 (52-55)	Immediate, delayed (2 mo)	NM (3) / NM	Mx, Md	JDEvolution (JDentalCare, Modena, Italy)	22 light smokers
**Grandi (5)**	2014	RCT (multicenter)	Italy / Private practice	25 (9/16)	39-74 (56)	Immediate	NM (1) / NM	Mx, Md	JDEvolution (JDentalCare, Modena, Italy)	9 light and heavy smokers
**Göthberg**	2016	RCT (unicenter)	Sweden / Public service	50 (18/32)	35-87 (67)	Immediate, delayed (3 mo)	II (3) / NM	Mx, Md	Brånemark TiUnite (Nobel Biocare, Göteborg, Sweden)	15 light smokers
**Han**	2018	PS (multicenter)	China / University	45 (17/28; 2)	26-73 (53)	Early (6 wk)	NM / NM	Mx, Md	OsseoSpeed TX (Dentsply, Mölndal, Sweden)	2 smokers<br>3 former smokers
**He**	2015	RA (unicenter)	China / University	1377 (704/673)	18-83 (44.9)	Immediate, delayed	NM	Mx, Md	Several (Straumann, Bego, Dentium, Anthogyr, Biomet 3i, Lifecore, Osstem, BLB)	Yes, but exact number not informed
**Higuchi**	2020	PS (multicenter)	USA, Chile, Spain, Australia, Italy / University + Private practice	110 (50/60; 8)	NM (61.7)	Immediate, early (within 10 d)	NM (14) / Yes	Md	Nobel Active (Nobel Biocare, Göteborg, Sweden)	23 smokers<br>47 former smokers
**Ji**	2012	RA (unicenter)	USA / University	45 (18/27)	25-88 (61.5)	Immediate	NM (1) / NM	Mx, Md	TiUnite, Steri-Oss (Nobel Biocare, Göteborg, Sweden), Screw-Vent (Zimmer Dental, Carlsbad, USA), XiVe (Dentsply Friadent, Mannhein, Germany)	8 smokers
**Kappel**	2016	RCT (unicenter)	Germany / University	46 (34/12)	NM (69.4)	Immediate	II (12) / NM	Md	Semados (Bego Implant Systems, Bremen, Germany)	2 light smokers<br>3 heavy smokers<br>3 extra-heavy smokers
**Keller**	1999	RA	USA / non-	54 (NM)	15-73	NM	II (2) / NM	Mx	Turned (Brånemark, Nobel	8 smokers

		(unicenter)	profit organizatio n		(28-59)				Biocare, Göteborg, Sweden)	12 former smokers
**Kim**	2018	RA (unicenter)	South Korea / University	881 (496/385)	17-90 (51.9)	Delayed	NM (68) / NM	Mx, Md	Tissue Level (Straumann, Basel, Switzerland)	NM
**Klotz**	2019	RA (unicenter)	Germany / Private practice	84 (28/56)	28-96 (60.2)	Delayed	NM / NM	Mx, Md	BlueSky (Bredent Medical)	Yes, but the exact number was not reported
**Koka**	2010	RA (unicenter)	USA / Non-profit organizatio n	137 (0/137)	50-93 (NM)	NM	NM (18) / NM	Mx, Md	NM	10 smokers
**Kourtis**	2004	RA (multicenter)	Greece / Private practice	405 (171/234)	18-83 (54.3)	Immediate, delayed	NM / NM	Mx, Md	Several (IMZ, Frialit-2, Freehex, Frialoc)	201 smokers
**Krennmair**	2013	RA (unicenter)	Austria / University	38 (NM)	NM (67.1)	Delayed (2-3 mo)	NM (4) / Yes	Md	Screw-Line Promote (Camlog Biotechnologies, Basel, Switzerland)	7 light smokers
**Krennmair (2)**	2016	CCT (unicenter)	Austria / University	41 (21/20)	NM (62.9)	Delayed (2 mo)	II (4) / Yes	Md	Camlog Screw-line (Promote+, Wimsheim, Germany)	5 light smokers
**Krennmair**	2019	PS (unicenter)	Austria / Private practice	85 (39/46)	NM (56.7)	Delayed (5-7 mo)	II (7) / Yes	Mx	Camlog Screw-line (Promote+, Wimsheim, Germany)	15 smokers
**Le**	2013	RA (unicenter)	USA / Private practice	168 (74/94)	34-87 (61)	NM	I (3) and II (15) / NM	Mx, Md	SLA (Straumann, Waldenburg, Switzerland), NM (AstraTech AB, Mölndal, Sweden), NM (Zimmer Dental, Warsaw, USA), NM (3i Implant Innovations, Palm Beach Gardens, USA), NM (BioHorizons, Birmingham, USA)	13 smokers
**Lee**	2019	RA (unicenter)	South Korea /	156 (70/86)	19-84 (59.9)	Delayed (mean 6.5	NM (18) / NM	Mx, Md	Several (Straumann, Dentium, Osstem, Astra Tech, Shinhung)	NM

			University			mo)				
**Levin**	2011	CCT (unicenter)	Israel / Private practice	717 (273/444)	NM (51)	NM	NM (81) / NM	Mx, Md	NM	103 smokers
**Lobato**	2020	RCT (unicenter)	Brazil / University	44 (NM)	25-77 (50.8)	Delayed (4-6 mo)	NM	Mx, Md	Alvim, Drive, and Titamax (Neodent, Curitiba, Brazil)	4 light smokers 3 heavy smokers
**Loo**	2009	CCT (unicenter)	China / University	278 (119/159)	38-50 (45.5)	Loading was not applied	II (138) / Yes	Mx, Md	NM (ITI, Straumann, Waldenburg, Switzerland)	NM
**Malchiodi**	2016	RCT (unicenter)	Italy / University	40 (24/16)	35-75 (52)	Delayed (3 mo)	NM (1) / NM	Mx, Md	SybronPRO XR (Sybron Implant Solutions)	10 light and heavy smokers
**Maló**	2011	RA (unicenter)	Portugal / Private clinic	245 (96/149)	23-85 (59)	Immediate	II (5) / NM	Md	Turned and TiUnite Brånemark (Nobel Biocare, Göteborg, Sweden)	61 smokers
**Maló**	2016	RA (unicenter)	Portugal / Private practice	721 (299/422)	20-87 (51)	Immediate, early, delayed	I (4) and II (52) / NM	Mx, Md	Mk II, Mk III, Mk IV, TiUnite, NobelSpeedy, NobelReplace (Nobel Biocare, Göteborg, Sweden)	477 smokers
**Maló (1)**	2019	RA (unicenter)	Portugal / Private practice	1072 (442/630; 40)	20-88 (55.8)	Immediate	NM (49) / NM	Mx	MK III, MK IV, NobelSpeedy Groovy (Nobel Biocare, Göteborg, Sweden)	241 smokers
**Maló (2)**	2019	RA (unicenter)	Portugal / Private practice	471 (185/286; 5)	20-85 (57.7)	Immediate	NM (11) / NM	Md	MK II, MK III, MK IV, NobelSpeedy Groovy (Nobel Biocare, Göteborg, Sweden)	117 smokers
**Mijiritsky**	2013	RA (multicenter)	NM / Private practice	787 (NM)	18-86 (53.7)	Immediate, delayed	NM	Mx, Md	NM (Adin Dental Implants, Alon Tavor, Israel)	Yes, but exact number not informed
**Morales-Vadillo**	2013	RA (unicenter)	Brazil / University	154 (NM)	20-87 (55.2)	Delayed	NM	Mx, Md	Bioform (BiomacMed, Juiz de Fora, Brazil)	141 smokers
**Morris**	2000	CCT (multicenter)	USA / Public service	663 (NM)	NM	NM	II (NM) / NM	Mx, Md	Turned (Spectra System, Core-Vent Corporation, DBA Paragon Company, Encino, USA), HA-coated (Spectra System, Core-Vent Corporation, DBA Paragon	NM

								Company, Encino, USA)	
**Niedermaier**	2017	RA (unicenter)	Germany / Private practice	380 (188/192)	23-92 (61.9)	Immediate	NM (25) / NM	Mx, Md	NanoTite (Biomet 3i, Palm Beach Gardens, USA), Nobel Active, Nobel Speedy, Nobel Speedy Replace and Nobel Replace CC (Nobel Biocare, Göteborg, Sweden)	141 smokers
**Nogueira**	2018	PS (unicenter)	Brazil / University	45 (11/34)	48-80 (63.4)	Delayed (3 mo)	NM (6) / NM	Md	Titamax (Neodent, Curitiba, Brazil)	5 smokers<br>18 former smokers
**Norton**	2017	PS (unicenter)	United Kingdom / Private practice	22 (10/12)	22-79 (NM)	Immediate	II (3) / NM	Mx, Md	Astra Tech AV (Dentsply, Mölndal, Sweden)	1 light smoker
**Omran**	2015	RA (unicenter)	USA / University	NM	≥18	NM	NM	Mx, Md	Several (Astra Tech Osseospeed, Biomet 3i, NobelReplace)	Yes, but exact number not informed
**Park**	2020	RA (unicenter)	South Korea / University	178 (87/91)	19-86 (53)	Delayed	NM (?) / NM	Mx	Several (Nobel Biocare, Straumann, Astra Tech, Dentium, Osstem, Shinheung)	NM
**Ravida**	2018	RA (unicenter)	USA / University	45 (24/21)	22-83 (58.9)	Immediate, delayed	NM (5) / No	Mx, Md	Several	5 smokers
**Ravida**	2019	RA (unicenter)	USA / University	145 (64/81)	NM (69.7)	NM	NM (10) / No	Mx, Md	NM	39 smokers
**Romandini**	2019	RA (unicenter)	Italy / University	52 (24/28)	NM (68.5)	NM	NM (9) / NM	Mx, Md	Several (Straumann, Biomet 3i, Nobel Biocare, Camlog)	10 smokers<br>4 former smokers
**Romero**	2020	RA (unicenter)	Spain / University	48 (26/22)	60-82 (68.6)	Early (6 wk)	NM (24) / NM	Md	Galimplant (Galimplant, Sarria, Spain)	22 smokers
**Rosen**	2018	RA (unicenter)	USA / Private practice	75 (27/48)	29-88 (61)	Delayed (2-6 mo)	NM (4) / NM	Mx, Md	ProActive (Neoss Ltd., Harrogate, England)	6 smokers
**Saridakis**	2018	RA (unicenter)	Germany / University	98 (NM)	> 18	Immediate, delayed	NM (5) / Yes	Mx, Md	Nobel Active (Nobel Biocare, Göteborg, Sweden)	No
**Schoenbaum**	2021	RA (multicenter)	USA / Private practice	378 (181/197)	NM (60)	NM	NM (NM) / NM	Mx, Md	NM	15% of the implants in smokers and former smokers

**Schwartz-Arad**	2016	RA (unicenter)	Israel / NM	214 (35/179)	NM (50.3)	Delayed (4-6 mo)	NM	Mx, Md	Several (Screw-Vent and Spline, Zimmer Dental Inc., Warsaw, USA; NobelActive and Replace Select, Nobel Biocare, Göteborg, Sweden; Implant Direct, Implant Direct LLC, Zurich, Switzerland)	39 smokers
**Shibuya**	2012	RA (unicenter)	Japan / University	9 (8/1)	22-71 (50.6)	NM	NM (2) / NM	Mx	TiUnite (Nobel Biocare, Göteborg, Sweden)	5 smokers
**Sicilia**	2021	RA (unicenter)	Spain / Private practice	268 (NM)	NM	Delayed (8-10 wk)	NM (11) NM	Md	Turned (Lifecore Biomedical), Osseotite (Biomet 3i), TiUnite (Nobel Biocare, Göteborg, Sweden)	75 smokers
**Simons**	2015	RA (unicenter)	Belgium / University	185 (69/116)	20-88 (56.4)	Delayed (3-6 mo)	NM (3) / NM	Md	Brånemark MK III (Nobel Biocare, Göteborg, Sweden)	29 smokers
**Souza**	2019	RA (unicenter)	Brazil / NM	10 (4/6)	49-70 (60)	Delayed (6 mo)	NM (2) / NM	Mx	Master Porous (Conexão Sistemas de Prótese Ltda, São Paulo, Brazil)	1 smoker
**Stacchi**	2021	RA (multicenter)	Italy / Private practice	156 (61/95)	22-86 (60.9)	NM	NM (3) / Yes	Mx	NM	29 smokers
**Tattan**	2021	RA (unicenter)	USA / University	201 (95/106)	45-69 (60)	NM	NM (18) / NM	Mx, Md	NM	37 smokers
**Tawil**	2008	CCT (unicenter)	Lebanon / University	90 (57/33)	29-85 (62)	Immediate, Delayed	II (45) / Yes	Mx, Md	Turned and TiUnite (Brånemark, Nobel Biocare, Göteborg, Sweden)	40 smokers
**Temmerman**	2015	PS (unicenter)	Belgium / University	28 (3/25)	42-76 (63)	Delayed (mean 3.6 mo)	NM (1) / NM	Mx, Md	NM (Astra Tech, Dentsply Implants, Mölndal, Sweden)	4 smokers
**Troiano**	2021	RA (unicenter)	Italy / Private practice	109 (63/46)	NM (58.1)	Immediate, Delayed (2-4 mo)	NM (NM) / Yes	Mx, Md	Several (3i, JD Dental Care, Astra Tech, Sweden & Martina, SIN, Henry Schein Krugg, Biosfera)	31 smokers
**van Steenberg**	2002	CCT (unicenter)	Belgium / University	399 (NM)	15-80 (50)	NM	I (NM) and II (NM)	Mx, Md	Turned (Brånemark, Nobel Biocare, Göteborg, Sweden)	Light and heavy smokers, but exact

**he**										number not informed
**Wang**	2020	RCT (unicenter)	USA / University	49 (15/34)	25-70 (46.8)	Immediate, Delayed (3 mo)	NM (2) / Yes	Mx, Md	NobelParallel Conical Connection (Nobel Biocare, Göteborg, Sweden)	1 light smoker
**Werbelow**	2020	RA (unicenter)	Germany / Private practice	23 (13/10)	42-74 (64)	Immediate	NM (3) / NM	Mx, Md	blueSKY© (Bredent GmbH & Co. KG, Senden, Germany)	2 smokers
**Zumstein**	2016	RA (unicenter)	Switzerland / Private practice	50 (22/28)	NM (58.6)	Immediate, delayed	II (1) / NM	Mx, Md	Proactive (Neoss Ltd, Harrogate, UK)	4 heavy smokers

NM – not mentioned; RA – retrospective analysis; PS – prospective study; CCT – controlled clinical trial; RCT – randomized controlled trial
d – days; wk – weeks; mo – months; Mx – maxilla; Md – mandible
[a] light smokers <10 cig/day; heavy smokers 10-20 cig/day; extra-heavy smokers >20 cig/day

d. Table S2. Quality assessment of the included studies, according to the National Institutes of Health (NIH).

Study	Year	Was the study question or objective clearly stated?	Was the study population clearly and fully described, including a case definition?	Were the cases consecutive?	Were the subjects comparable?	Was the intervention clearly described?	Were the outcome measures clearly defined, valid, reliable, and implemented consistently across all study participants?	Was the length of follow-up adequate? [a]	Were the statistical methods well-described?	Were the results well-described?	Total (n/9)
Accursi	2000	1	1	1	1	1	1	1	1	1	9/9
Aguilar-Salvatierra	2016	1	1	0	1	1	1	1	0	1	7/9
Alqahtani	2020	1	1	0	1	1	1	1	0	1	7/9
Alsaadi (1)	2007	1	1	1	1	1	1	1	1	1	9/9
Alsaadi (2)	2008	1	1	0	1	1	1	1	1	1	8/9
Al-Sabbagh	2015	1	1	0	1	1	1	1	1	0	8/9
Alsahhaf	2019	1	1	0	1	1	1	1	0	1	7/9
Altay	2018	1	1	0	1	1	1	1	0	1	7/9
Anner	2010	1	1	0	1	1	1	1	0	1	7/9
Atarchi	2020	1	1	0	1	1	1	1	1	1	8/9
Bell	2011	1	1	0	1	1	1	1	0	1	7/9
Boardman	2016	1	1	0	1	1	1	1	1	1	7/9
Boboeva	2021	1	1	1	1	1	1	1	1	1	9/9
Cabrera-Dominguez	2017	1	1	0	1	1	1	1	0	1	7/9
Cannizzaro	2003	1	1	0	1	1	1	1	0	1	7/9
Chang	2020	1	1	0	1	1	1	1	1	1	8/9
Chrcanovic	2016	1	1	1	1	1	1	1	1	1	9/9
Clauser	2020	1	1	1	1	1	1	1	1	1	9/9
Coskunses	2021	1	1	1	1	1	1	1	1	1	9/9
Daneshvar	2016	1	1	1	1	1	1	1	0	1	8/9
Daubert	2015	1	1	1	1	1	1	1	1	1	9/9

Dhanrajani	2005	1	1	1	1	1	1	1	0	1	8/9
Dowell	2007	1	1	0	1	1	1	1	0	1	7/9
Doyle	2007	1	1	0	1	1	1	1	0	1	7/9
Erdogan	2015	1	1	0	1	1	1	1	1	1	8/9
Feher	2020	1	1	0	1	1	1	1	1	1	8/9
French	2015	1	1	0	1	1	1	1	1	1	8/9
Gherlone	2016	1	1	0	1	1	1	1	1	1	8/9
Ghiraldini	2016	1	1	0	1	1	1	1	1	1	8/9
Gjelvold	2020	1	1	1	1	1	1	1	1	1	9/9
Gómez-Moreno	2015	1	1	0	1	1	1	1	0	1	7/9
Grandi (1)	2012	1	1	1	1	1	1	1	1	1	9/9
Grandi (2)	2012	1	1	0	1	1	1	1	1	1	8/9
Grandi (3)	2012	1	1	1	1	1	1	1	1	1	9/9
Grandi (4)	2013	1	1	1	1	1	1	1	1	1	9/9
Grandi (5)	2014	1	1	0	1	1	1	1	1	1	8/9
Göthberg	2016	1	1	0	1	1	1	1	1	1	8/9
Han	2018	1	1	0	1	1	1	1	1	1	8/9
He	2015	1	1	1	1	1	1	1	1	1	9/9
Higuchi	2020	1	1	0	1	1	1	1	0	1	7/9
Ji	2012	1	1	0	1	1	1	1	0	1	7/9
Kappel	2016	1	1	0	1	1	1	1	1	1	8/9
Keller	1999	1	1	1	1	1	1	1	0	1	8/9
Kim	2018	1	1	1	1	1	1	1	1	1	9/9
Klotz	2019	1	1	1	1	1	1	1	1	1	9/9
Koka	2010	1	1	0	1	1	1	1	0	1	7/9
Kourtis	2004	1	1	1	1	1	1	1	0	1	8/9
Krennmair (1)	2013	1	1	0	1	1	1	1	0	1	7/9
Krennmair (2)	2016	1	1	0	1	1	1	1	1	1	8/9
Krennmair (3)	2019	1	1	1	1	1	1	1	1	1	9/9
Le	2013	1	1	0	1	1	1	1	0	1	7/9
Lee	2019	1	1	0	1	1	1	1	1	1	8/9

Levin	2011	1	1	1	1	1	1	1	1	1	9/9
Lobato	2020	1	1	0	1	1	1	1	1	0	7/9
Loo	2009	1	1	0	1	1	1	1	0	1	7/9
Malchiodi	2016	1	1	0	1	1	1	1	1	1	8/9
Maló (1)	2011	1	1	1	1	1	1	1	0	1	8/9
Maló (2)	2016	1	1	0	1	1	1	1	1	1	8/9
Maló (3)	2019	1	1	1	1	1	1	1	1	1	9/9
Maló (4)	2019	1	1	0	1	1	1	1	1	1	8/9
Mijiritsky	2013	1	1	1	1	1	1	1	0	1	8/9
Morales-Vadillo	2013	1	1	1	1	1	1	1	0	1	8/9
Morris	2000	1	1	0	1	1	1	1	0	1	7/9
Niedemaier	2017	1	1	0	1	1	1	1	1	1	8/9
Nogueira	2018	1	1	1	1	1	1	1	1	1	9/9
Norton	2017	1	1	1	1	1	1	1	1	1	9/9
Omran	2015	1	1	1	1	1	1	1	1	1	9/9
Park	2020	1	1	0	1	1	1	1	1	1	8/9
Ravida (1)	2018	1	1	1	1	1	1	1	1	1	9/9
Ravida (2)	2019	1	1	1	1	1	1	1	1	1	9/9
Romandini	2019	1	1	0	1	1	1	1	0	1	7/9
Romero	2020	1	1	0	1	1	1	1	0	1	7/9
Rosen	2018	1	1	1	1	1	1	1	1	1	9/9
Saridakis	2018	1	1	0	1	1	1	1	1	1	8/9
Schoenbaum	2021	1	1	0	1	1	1	1	1	1	8/9
Schwartz-Arad	2016	1	1	1	1	1	1	1	1	1	9/9
Shibuya	2012	1	1	0	1	1	1	1	0	1	7/9
Sicilia	2021	1	1	1	1	1	1	1	1	1	9/9
Simons	2015	1	1	0	1	1	1	1	1	1	8/9
Souza	2019	1	1	0	1	1	1	1	0	1	7/9
Stacchi	2021	1	1	1	1	1	1	1	1	1	9/9
Tattan	2021	1	1	1	1	1	1	1	1	1	9/9
Tawil	2008	1	1	1	1	1	1	1	1	1	9/9
Temmerman	2015	1	1	0	1	1	1	1	1	1	8/9
Troiano	2021	1	1	0	1	1	1	1	1	1	8/9

van Steenberghe	2002	1	1	1	1	1	1	1	0	1	8/9
Wang	2020	1	1	0	1	1	1	1	1	1	8/9
Werbelow	2020	1	1	1	1	1	1	1	1	1	9/9
Zumstein	2016	1	1	1	1	1	1	1	1	1	9/9

[a] 3 months of follow-up was chosen to be of adequate length.
